# Building Trust Across Borders? Exploring the Trust-Building Process Between the Nonprofit Organizations and the Government in China

**DOI:** 10.3389/fpsyg.2020.582821

**Published:** 2021-01-20

**Authors:** Ying Xu

**Affiliations:** Department of Sociology, Law School, Shenzhen University, Shenzhen, China

**Keywords:** NPO, trust, moral resource, political capital, government, China

## Introduction

According to Sztompka (1996), “trust is a bet on the future contingent action of others” (p. 39). Sometimes trust may be treated as “a psychological trait” at the individual level. However, without doubt, trust can be “shared by a number of individuals” in a certain society (Sztompka, 1998, p. 20). In this perspective, trust is considered at the societal level, which includes, but reaches beyond, the attitudes of individuals. And it is believed that “when there is trust there are increased possibilities for experience and action” (Luhmann, 2017, p. 8); “a nation's well-being, as well as its ability to compete,” is conditioned on “a single, pervasive cultural characteristic: the level of trust inherent in a society” (Fukuyama, 1995, p. 7).

It has been found that the democratic order has a significant trust-generating force because establishing a set of universal criteria that regulates the institutions of both government and civil society contributes to generating social trust among citizens (Sztompka, 1996; Bielefeld, 2006). However, little research has been done to explore the process and mechanism of trust building in countries that have not set up a democratic order. To fill the research gap, this study aims to explore the trust building process among nonprofit organizations (NPOs), the government and service users in a changing China.

## Moral Resources, Political Capital, and Trust-Building

The Chinese government has cautiously welcomed NPOs participating in the area of social service delivery since the market-oriented reforms launched in 1978 (Xu and Ngai, 2011; Xu, 2016). On the one hand, a centralist cultural heritage champions authorities and collective values. According to the 2020 Edelman Trust Barometer, Chinese people's trust in the government and NGOs is very high, ranking first and second among 26 global markets, respectively (Edelman, 2020). On the other hand, misunderstandings or distrust occur from time to time between the Chinese government and NGOs/NPOs or so-called “civil society” (Evans, 2010; Zhou, 2011). Under such circumstances there is a need to understand how the trust-building capacity of NPOs is culturally and politically bound and how to improve their capacity.

The moral resources and political capital perspective provides valuable information for grassroots NPOs on how to build trust with the government in China (Xu and Ngai, 2011; Xu, 2013). And previous studies have indicated that the NPOs that are involved in social service delivery are repositories of moral resources and therefore likely to advance trust (Xu, 2013). Moral resources refer to the available moral choices that could be made by any organization. There are two types of moral resources: (1) self-chosen moral resources-I, which are rooted in Immanuel Kant's (1998) argument of “What ought I to do?;” and (2) societally recognized moral resources-II, which follow Adorno's (2000) argument that moral or immoral tropes are socially determined (Xu and Ngai, 2011; Xu, 2013).

Due to the centralized political tradition, political capital is very important for NPOs in gaining trust from the government (Xu, 2013). Political capital means the capital that will improve or enhance the organizations' status, assets or access in the existing political system. There are two types of political capital: (1) ascribed political capital-I, which refers to the political status that is conferred upon certain organizations through historical inheritance, and (2) achieved political capital-II, which refers to the political resources achieved by the organizations' own efforts (Blau and Duncan, 1967; Xu and Ngai, 2011).

It was found that possessing the “societally recognized moral resources-II” is very important for grassroots and foreign organizations which have little ascribed political capital-I, because moral resources-II may help organizations to gain the trust of the public and the government, which may enable them to build political capital-II. In this sense, the NPOs aiming to provide social services—which usually focus on the common good and therefore possess moral resource-II—have a promising future with regards to improving trust-building between heterogeneous groups (Xu and Ngai, 2011).

It is worth noting trust relationships are vulnerable as trust can be withdrawn from objects which have previously been trusted. For example, when “gifts” accepted by officials or medical doctors secure favors or preferential treatment, both institutional trust and positional trust may be destroyed by the bribe givers and bribe takers (Sztompka, 1996; Heimer, 2001). In this regard, ethics rules such as non-distribution constraint, which allows NPOs to make profits but prevents them from distributing them to private parties, are crucial factors in improving the trustworthiness of NPOs and convincing an increasingly skeptical public (Hansmann, 2003; Becker, 2018; Vaceková and Plaček, 2020). Therefore, the trust-building process demands a broad and comprehensive perspective (Becker et al., 2019) and the relationship between NPOs and stakeholders (e.g., the government and service users) needs to be further investigated.

## Revisiting the Dimensions of trust

Trust can be studied at different levels. First, at the individual level, interpersonal trust can be described as a three-part relation: “A trusts B to do X” (Hardin, 2001, p. 14). Yet, who is the B, what is the X, and how can a trusting relationship develop? From a cognitive perspective, scholars believe that trust is grounded in the moral commitments of the trusted (Messick and Kramer, 2001). In other words, “A trusts B” because A knows that B has strong moral commitments to live up to certain trust expectations that A places in B (Messick and Kramer, 2001). But how can A believe that B will follow the ethical rule? Furthermore, in reality, even if A knows that B is a very honest person, A might trust B to manage her/his money but not trust B to take care of her/his baby. An answer to this question is that the truster's belief derives from experience, which means that B's qualities or previous behaviors convince A to trust B.Moreover, based on the cognitive assessment, studies have verified that if one's experience with others (especially in one's early years) has been good and cooperative, then one tends to trust others (Yamagishi, 2001). In this sense, trust is not only a matter of knowledge or belief in somebody or something, but also a learning process that can be measured through behavior (Hardin, 2001; Yamagishi, 2001). Empirical studies of trust have divided people into those high trusters, who are more likely to trust strangers, and low trusters, who are likely to distrust others. The concept of generalized trust refers to the observation that “some people have a greater psychological disposition to trust than others do,” is developed (Hardin, 2001, p. 15).

Hence, when a behavioral measure is adopted to assess generalized trust, the accounts of trust go beyond interpersonal relationships and extend into the social realm (such as the NPOs), because both the behavior and the generalized trust are significantly related to a particular social context with its norms, its legal environment, and its local culture (Sztompka, 1998).

Second, at the intra-group level, evidence showed that the generalized trust that is primarily based on membership networks may facilitate more positive interactions within certain types of associations. However, it is doubtful that the network-based trust can be generalized to strangers in the society concerned (Stolle, 2001). Similarly, many scholars have regarded kin-like relationships as one of the most important social bases of trust. For instance, Cook and Cook and Hardin (2001) argued that it is “familial, communal, network, and other contexts” that are grounds for trust in the people we might trust (p. 330). Particularly, Ensminger (2001) conducted a case study of East African herders, and found that kin relationships and reputation are significant bases for trust. In Europe, by analyzing the data of Eurobarometer surveys undertaken during 1980–1996, Mackie (2001) pointed out that Europeans are likely to regard people of their own country as more trustworthy than the people of other countries. Moreover, it is found that differing patterns of family formation may have been a significant basis for the development of trust (Mackie, 2001). However, as Fukuyama (1995) argued, for instance, although Chinese Confucianism promotes tremendous trust in the family setting, social trust outside the family is relatively low.

Third, consideration should also be given to the institutional level. Because intra-group trust based on kinships or association memberships can fail to develop into generalized trust because of inter-group conflicts of interests, Fukuyama (1995) and Knight (2001) presumed that formal institutions (such as the state and the law) should provide assurance to improve the trust across boundaries. Moreover, it was assumed that good governance implies a mutual trust between citizens and governors and among the fellow citizens (Levi and Braithwaite, 1998). Thus, this kind of trust is inherently institutional in nature because any reference to “the fellow citizens” refers to a generic category of “everyone else” rather than one's “neighbours” (Offe, 1999).

Last but not least, “trusting an institution” means that the citizens are confident that the institution will continue to operate according to the established rules in the way that the citizens have known (Offe, 1999). The level of institutional trust may be positively associated with the level of positional trust, which means the trust of people because they hold certain positions such as lawyers, teachers, doctors, social workers or other professionals (Giddens, 1990; Sztompka, 1996).

## Conclusion

**Figure 1:**
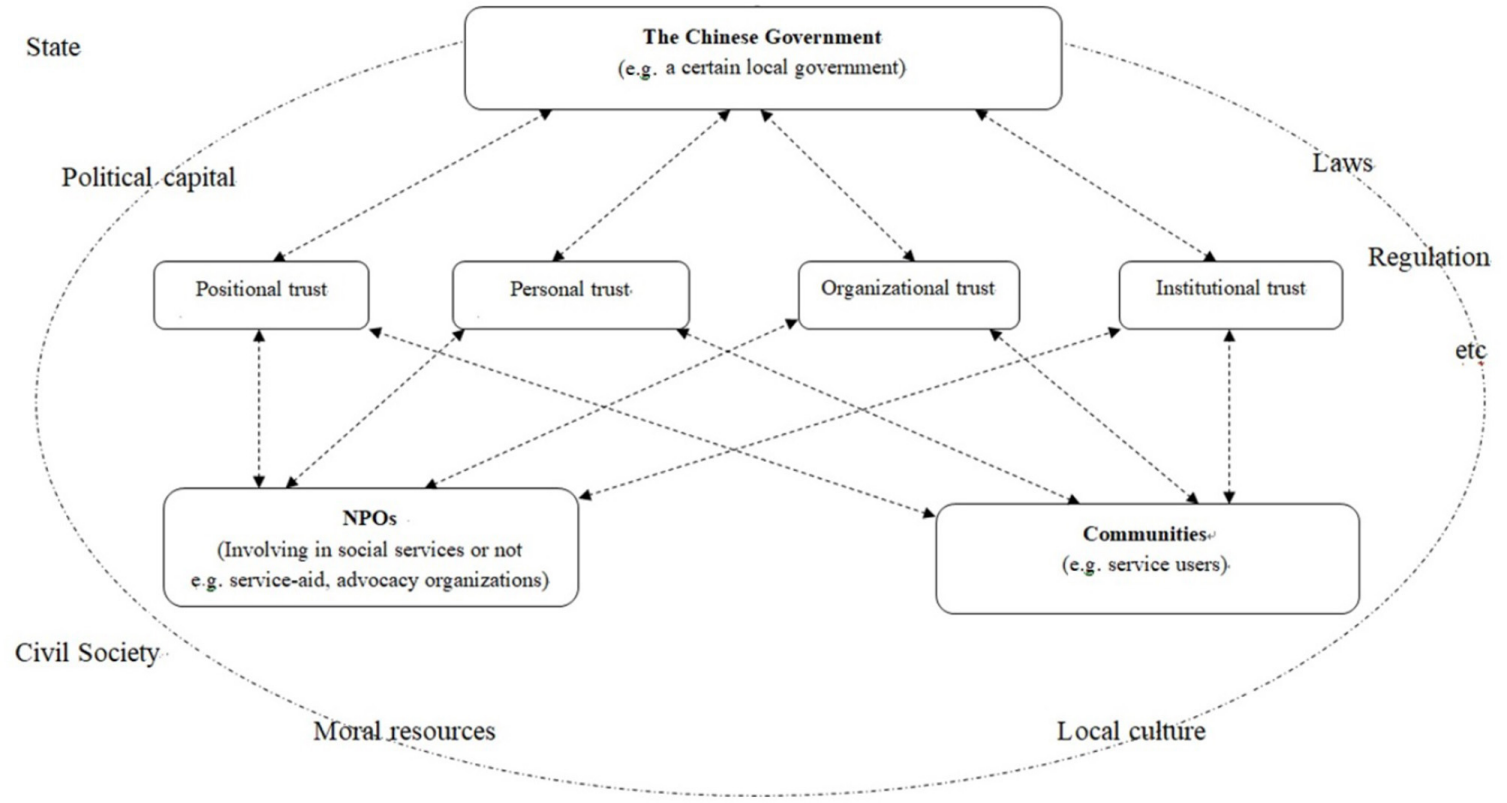
A research framework for trust-building.

Based on the discussion above, a research framework for trust-building, which explores personal, positional, organizational and institutional trust between the NPOs and stakeholders in China is developed (Figure 1). The key terms and factors are operationally defined as follows.

First, the process of trust-building will be analyzed from a perspective of four dimensions (Giddens, 1990; Sztompka, 1996):

1) Personal trust, which means the trust between individuals;
2) Positional trust, which means the trust of a person in those in certain positions such as lawyers, teachers, doctors, social workers or other professionals;
3) Intra-group trust, which means network-based trust, such as kin relationships or small community-based local organizations;
4) Institutional trust, which means the trust in the institutional systems of a society, such as the education, medical or judicial systems, and so on.

Second, trust-building, which refers to the process of building trust, “is also a kind of—socially objectified... cultural capital from which individuals can draw in their actions” (Sztompka, 1998, p. 20). In contrast to organizations that mainly pursue their own local political or economic objectives, NPOs committed to social service projects are likely to gain support from certain communities and are thus repositories of moral resources, and could be an important basis for building trust with the government (Fenton et al., 1999; Halfpenny, 2000; Lee et al., 2014; Feng, 2017). In other words, moral resources and local culture that embody the communities and civil society and political capital, laws, and/or regulations that are associated with the state jointly affect the trust among NPOs, the government, and service users.

Particularly in countries where laws and regulations are relatively weak, gaining political capital would increase governments' trust in NPOs other than social service organizations (Xu, 2013, 2016). In recent years, NPOs began to use social media (e.g., WeChat, QQ, Twitter, etc.) to disseminate information, build engagement, and facilitate action (Guo and Saxton, 2014; Svensson et al., 2015). These new Information and Communication Technologies (ICTs) have allowed the NPOs' efforts to be more easily heard and seen by the public. They have provided new opportunities for various NPOs to get societally recognized moral resources-II and develop their achieved political capital-II, which may facilitate the trust-building process between them and the government (Xu, 2014).

In short, research increasingly suggests that trust is a major factor in the success of effective collaboration among institutions. This study improves theoretical understanding of key factors that may facilitate, and/or hinder, the building of trust. I hope that the further research may develop practical suggestions for improving trust-building, and thus contribute to the welfare and success of NPOs, service users and the Chinese government.

## Author Contributions

The author confirms being the sole contributor of this work and has approved it for publication.

## Conflict of Interest

The author declares that the research was conducted in the absence of any commercial or financial relationships that could be construed as a potential conflict of interest.
